# Autophagy: an affair of the heart

**DOI:** 10.1007/s10741-012-9367-2

**Published:** 2012-11-28

**Authors:** Roberta A. Gottlieb, Robert M. Mentzer

**Affiliations:** 1 Donald P. Shiley BioScience Center, San Diego State University, San Diego, CA USA; 2 WSU Cardiovascular Research Institute, Wayne State University School of Medicine, Detroit, MI USA; 3 Departments of Physiology and Surgery, Wayne State University School of Medicine, Detroit, MI USA

**Keywords:** Autophagy, Mitophagy, Cardioprotection, Hypertrophy, Heart failure

## Abstract

Whether an element of routine housekeeping or in the setting of imminent disaster, it is a good idea to get one’s affairs in order. Autophagy, the process of recycling organelles and protein aggregates, is a basal homeostatic process and an evolutionarily conserved response to starvation and other forms of metabolic stress. Our understanding of the role of autophagy in the heart is changing rapidly as new information becomes available. This review examines the role of autophagy in the heart in the setting of cardioprotection, hypertrophy, and heart failure. Contradictory findings are reconciled in light of recent developments. The preponderance of evidence favors a beneficial role for autophagy in the heart under most conditions.

## Introduction

It is becoming increasingly apparent that autophagy and mitophagy play an important role in modulating the cell’s response to stress in a number of organs including brain, liver, skeletal muscle, pancreas, and heart. This review will focus on cardiac autophagy; the purpose is to summarize the role of autophagy in homeostasis and pathophysiology in the adult heart, and where possible, reconcile the studies that suggest cardiac autophagy is a maladaptive process as opposed to an adaptive one. In addition to cardioprotection in the setting of ischemia–reperfusion, we will also examine autophagy and mitophagy with a particular emphasis on cardiac hypertrophy and heart failure.

## Autophagy, mitophagy, and cardioprotection

Autophagy is the cell-autonomous process of bulk recycling of protein aggregates; mitophagy is the specialized form of autophagy responsible for elimination of mitochondria. Early studies of cardiac autophagy noted that mitochondria were frequently contained within autophagosomes, but the significance of this was not established until much later. Studies of ischemia/reperfusion injury revealed that reactive oxygen species (ROS) and mitochondria were a critical target of injury, as catastrophic opening of the mitochondrial permeability transition pore (mPTP) commonly culminated in cell death. Many studies showed that preventing pore opening reduced infarct size, reinforcing the notion that mitochondrial injury was a critical event. Studies of mitochondria in isolated cardiomyocytes led to the recognition of ROS-induced ROS release, a self-propagating phenomenon in which ROS release from one mitochondrion could trigger ROS release and depolarization of adjacent mitochondria in a rapidly expanding pattern. The ability to propagate this behavior was increased if glutathione levels were decreased. Thus, it was recognized that under conditions of oxidative stress a single damaged mitochondrion might be sufficient to trigger a mitochondrial stampede to cell death.

### Ischemic preconditioning

The most potent and reproducible means to protect the heart from ischemic injury is ischemic preconditioning (IPC). In IPC, the heart is exposed to a brief ischemic stress prior to the index ischemia. There is general agreement that preconditioning converges on the mitochondria, triggering mild depolarization, which is thought to prevent calcium overload and mPTP opening. However, mild mitochondrial depolarization is also known to trigger mitophagy: PINK1 is constitutively degraded so long as mitochondrial membrane potential is high. However, when membrane potential drops, PINK1 accumulates and recruits Parkin, which ubiquitinates multiple outer mitochondrial membrane proteins and recruits the autophagic machinery.

Autophagy, which delivers cellular material to the lysosome for degradation, was presumed to provide fuel for energy production during ischemia; however, we now know that energy production may not be critical. We have suggested that the value of autophagy may lie in sequestering the most unstable mitochondria before they can set a stampede in motion. The evidence for this is based on the report that preventing lysosomal degradation does not affect infarct size in a Langendorff model of I/R injury, and on the key observation that inactivation of Parkin-dependent mitophagy abolishes ischemic preconditioning [1].

The study by Zoltan et al. showed that blocking initiation of autophagy with a cell permeable dominant negative Atg5 mutant (Tat-Atg5^K130R^) abolished the cardioprotective effects of the autophagy-inducing drug, chloramphenicol [2]. In contrast, blocking the degradative phase of autophagy with the lysosomotropic agent chloroquine did not interfere with cardioprotection mediated by chloramphenicol. This suggests that sequestration of material into autophagosomes is essential for cardioprotection, but liberation of breakdown products for energy production is not.

The study by Huang et al. showed that mitophagy mediated by Parkin was essential for cardioprotection. In that study, targets of autophagy—other than mitophagy—were not disrupted but cardioprotection was lost. The most reasonable interpretation (though not the only one) is that provision of metabolites, which presumably was not obstructed in the Parkin-null animals, was not as important as sequestration of mitochondria, a process that depended upon Parkin. This leads to the supposition that sequestration of damaged mitochondria will avert the propagation of ROS-induced ROS release and cell death. Therefore, any intervention that triggers mild mitochondrial depolarization and removal by mitophagy could be cardioprotective.

### Pharmacologic conditioning

Autophagy has been shown to be induced by a number of cardioprotective agents, including diazoxide, UTP, CCPA, ranolazine, rapamycin, sulfaphenazole, chloramphenicol, and statins [3]. Not all of these agents have been evaluated for their effects on mitochondrial membrane potential and mitophagy, but a few have. Agents that are known to trigger mild depolarization include those that open the mitochondrial ATP-sensitive potassium channel (mitoK_ATP_), such as diazoxide [4]; induction of uncoupling proteins [5–7]; volatile anesthetics, which act on BK_Ca_ [8]; low-dose FCCP [9]; and interventions which trigger flickering of the mPTP such as ischemic preconditioning [10]. These are all well-known cardioprotective agents which additionally share the property of causing mild mitochondrial depolarization. Not all of these agents have been evaluated for their ability to induce autophagy, but diazoxide and FCCP have been shown to do so, and starvation-induced autophagy is attenuated by inhibition or deletion of cyclophilin D, a key regulator of the mPTP. None of these agents have been assessed directly for their effects on Parkin translocation or mitophagy, with the exception of FCCP and ischemic preconditioning [1, 9]. Ischemic preconditioning triggered rapid translocation of Parkin to mitochondria, followed by mitophagy. Ischemic preconditioning was ineffective in Parkin-null mice, implicating Parkin-dependent mitophagy in the mechanism of cardioprotection.

### The adaptive versus maladaptive story

Controversy exists as to whether autophagy is always a good thing for the heart. Sadoshima’s group concluded that autophagy was beneficial during ischemia but deleterious during reperfusion [11]. However, they have not examined ischemic preconditioning or other cardioprotective interventions. Bnip3, which is activated during ischemia/reperfusion, triggers mitochondrial depolarization and mitophagy which is Parkin-dependent [12]. Bnip3, a pro-apoptotic member of the Bcl-2 family, causes mitochondrial damage; injury is exacerbated if autophagy is suppressed [13]. However, Beclin 1 haploinsufficiency was reported to decrease ischemia/reperfusion injury, an effect which was assumed to be due to diminished autophagy [11]. Partial knockdown of Beclin 1 or haploinsufficiency does not limit the initiation of autophagy, although a more complete knockdown does [14]. Beclin 1 interacts with autophagy machinery at the lysosomal fusion stage as well, and in that setting, it *delays* autophagosome-lysosome fusion. Reduction in the amount of Beclin 1 actually accelerates flux, resulting in fewer autophagosomes hanging around and the misleading appearance of less autophagy.

This conundrum was beautifully elucidated recently by Diwan’s group [15] and allows reinterpretation of the confusing results of Hill’s group who reported that autophagy contributed to maladaptive remodeling after severe TAC [16]. They based their conclusions on the presence of increased number of autophagosomes after banding, which was reduced in Beclin 1 haploinsufficient mice, and resulted in a better outcome than WT mice. If Diwan’s study is correct, then we can reinterpret the observations of Zhu et al. [16] to conclude that the increased autophagosome abundance in the pressure-overloaded animals is a reflection of compensatory autophagy that is somewhat ineffective due to Beclin 1-mediated impairment of flux. Reducing Beclin 1 accelerates flux and ameliorates remodeling. This makes sense for several reasons: (1) autophagy negatively regulates cell size, so increased autophagy should oppose hypertrophy; (2) autophagy is protective in other forms of heart disease such as desmin-related cardiomyopathy (aggregopathy) and ischemia/reperfusion injury; and (3) impaired autophagy is associated with increased inflammation in a wide variety of disorders, and inflammation contributes to most forms of cardiac pathology.

Still to resolve are the findings from Sadoshima’s group who have developed evidence that autophagy is beneficial during ischemia but harmful during reperfusion [11, 17]. Although initial studies also relied on Beclin 1 (±) mice, another study used AMPK-DN mice and reached similar conclusions. It is possible that autophagy or mitophagy is important for preconditioning or ischemia, but deleterious in reperfusion. In contrast, chloramphenicol and sulfaphenazole, which are potent cardioprotectants effective when given at reperfusion, act through induction of autophagy, suggesting that autophagy is beneficial at reperfusion [3, 18, 19]. More work will be required to reconcile Sadoshima’s findings and our own. While our studies have focused on Parkin as an important mediator of mitophagy in IPC, it is likely that it is not the only pathway. Degradation of individual mitochondrial proteins via mitochondrial proteases or the ubiquitin–proteasome system may proceed independently of bulk degradation via mitophagy in steady state conditions. Moreover, redistribution of mitochondrial proteins via fusion and fission further complicates the analysis of mitochondrial and protein turnover [20].

### Is mitophagy enough?

One might wonder whether eliminating mitochondria is such a good idea. After all, the heart needs lots of mitochondria to make ATP: can it afford to lose any? One argument is that mitochondria which are damaged and possibly uncoupled might actually consume ATP, so eliminating those slackers might actually improve ATP production efficiency. A second argument is that there may be excess capacity. According to R. Balaban [21], in large animals (dog, pig, human), ATP production in the resting heart is about 20 % of maximal capacity; thus, loss of some mitochondria will not limit cardiac ATP production until demand is quite high. Note that in mice, basal cardiac ATP production is already at 60 % of maximal capacity; thus, a loss of mitochondria might affect cardiac function with just a modest increase in work. The third and most appealing argument is that mitochondrial biogenesis may be upregulated to keep up with destruction.

It is recognized that statins induce PGC1alpha, the master regulator of mitochondrial biogenesis. Less is known about whether other cardioprotective agents also trigger biogenesis, but one hint is that a transcriptional inhibitor of PGC1alpha, PARIS, is a substrate of Parkin [22]. If PARIS spends time on mitochondria as well as in the nucleus, then Parkin-dependent mitophagy would also lower total levels of PARIS, leading to de-repression of PGC1alpha and upregulation of mitochondrial biogenesis. Finally, if mitophagy is too vigorous and ATP levels decline, then AMP-activated kinase (AMPK) will activate PGC1alpha. Thus, under normal conditions, homeostatic mechanisms will preserve mitochondrial content. This may be altered in type 2 diabetes mellitus (T2DM), where autophagy is diminished, and where PGC1alpha is also downregulated through modification of serine residues by N-acetyl-glucosamine (O-GlcNAc) [23]. It is possible that in this setting, if mitophagy is triggered, biogenesis may not be upregulated appropriately. Conceivably, this loss of homeostasis may demarcate the transition from compensated heart failure to decompensation.

Efforts in the coming years should shed light on the importance of homeostatic control of mitophagy and biogenesis. Even in settings where mitobiogenesis balances mitophagy, the rate of turnover could contribute to pathophysiology. Slow mitochondrial turnover may lead to accumulation of damaged and inefficient mitochondria which generate elevated levels of ROS, thereby driving inflammatory signaling, as recently reported by two independent groups [24, 25]. Otsu’s group showed that this contributed to adverse cardiac remodeling [24]. Our understanding of mitochondrial turnover will benefit from better tools to monitor the process. These will likely come from proteomics in which isotopic labeling will provide information about turnover of individual proteins, and from use of transgenic mice that will allow monitoring of mitochondrial turnover. Stay tuned for new developments in this rapidly expanding field. Interestingly, agents that accelerate mitochondrial turnover are all things we consider “good for us”, such as exercise, intermittent fasting or caloric restriction, and statins, whereas things that suppress autophagy (and mitophagy), including age, obesity, dyslipidemia, and insulin resistance, are associated with increased risk of adverse cardiac outcomes.

### Biogenesis, fission and fusion

Mitochondria are dynamic organelles—at least in cultured cell lines—that interact as part of an interconnected network at some times and that exist as solitary organelles at others. Key genes regulating mitochondrial dynamics (fusion and fission) have been identified, and mutations are associated with human disease, including Charcot-Marie-Tooth type 2 (mitofusin2 mutation) and optic atrophy (Opa1 mutation). Neither of these human mutations results in clinically apparent heart disease, raising questions as to the importance of mitochondrial fusion and fission in the heart. However, conditional knockout of mitofusin1 and mitofusin2 (mfn1/mfn2 DKO) results in development of heart failure over several weeks, suggesting that mitochondrial fusion events are important to cardiac homeostasis [26].

It appears that mitochondrial fusion events in the heart occur on a glacial time scale, if at all. Dorn estimated that a mitochondrial fusion/fission cycle in the heart occurs every 16 days. This is impossible to distinguish from mitochondrial turnover, as mitochondrial half-life in the heart is also about 2 weeks. Interestingly, a recent paper by Zhao et al. [27] proposes a role for Mfn2 in autophagosome/lysosome fusion. It is possible that this second function of Mfn2 is more important in the heart.

Finally, Scorrano has shown that Mfn2 is important for anchoring specialized portions of the ER to mitochondria [28]; this could be important for calcium regulation of mitochondrial energetics; loss of Mfn2 anchoring could lead to progressive derangement of calcium and energy homeostasis. Moreover, Ngoh et al. [29] reported that Mfn2 deletion amplified ER stress. Whether mitochondrial fusion and fission events occur in the heart is a subject of debate, as the tightly packed architecture of the cardiomyocyte would pose considerable physical barriers. Deletion of Mfn1 in the heart results in an increase in the number of small spherical mitochondria and greater resistance to hydrogen peroxide [30]. Deletion of both Mfn1 and Mfn2 results in cardiomyopathy and death by postnatal day 16, [31] suggesting an important role for mitofusins during early postnatal cardiac development.

Fusion/fission events are poorly understood in the context of ischemic preconditioning or ischemia/reperfusion injury. One study using mDivi1, an inhibitor of the fission protein Drp1, concluded that inhibiting fission in the context of I/R injury was protective [32]. However, further evidence is needed to confirm that mDivi1 was acting through inhibition of mitochondrial fission. The benefit of acute administration of a fission inhibitor is inconsistent with the exceedingly slow kinetics of fusion events in the heart. Thus, it is likely that Drp1 has other functions (e.g., interaction with apoptosis machinery), mDivi1 has other targets, or that fusion and fission events in the heart occur much more rapidly under stress conditions. More work is needed to understand the role of fission and fusion machinery in the heart.

## Autophagy and cardiac remodeling

Heart failure is a leading cause of death in the North America and Europe. It is a clinical syndrome characterized by a decline in cardiac output, inadequate systemic perfusion, and manifestations of pulmonary congestion and peripheral vasoconstriction. Therapeutic interventions are designed to improve contractility of the heart and reduce peripheral arterial resistance. Despite the use of angiotensin converting enzyme (ACE) inhibitors, beta blockers, vasodilators, and diuretics, however, mortality remains high and approaches 50 % at 5 years [33]. In order to develop more effective therapies directed toward both prevention and treatment, a much better understanding of the pathology that leads to heart failure is needed.

The most commonly encountered pathologies that progress to heart failure are ischemic and load-induced remodeling. Ischemic remodeling occurs after a myocardial infarction; load-induced remodeling occurs in the setting of hypertension (pressure overload), valvular dysfunction (pressure or volume overload), and intrinsic contractile abnormalities (certain cardiomyopathies). While both involve multiple complex signaling pathways and neural-humoral interactions, there is evidence that remodeling also involves autophagy and mitophagy. It is controversial, however, as to whether, in the context of remodeling, these processes are adaptive or maladaptive.

### Ischemic remodeling

The mechanisms involved in post-infarction cardiac remodeling are multi-factorial and include neuro-humoral imbalance, inflammation, and apoptosis [34]. It is defined by infarct expansion and acute dilatation of the infarct zone, dilatation of noninfarct zone and increased sphericity, and ventricular hypertrophy. The end result is progressive deterioration in left ventricular function [35]. The process is complicated, and many factors are associated with disease progression including phosphoinositide-3-kinase (PI3K), mitogen-activated protein kinase (MAPK) pathways, altered extracellular matrix, and expression of various cytokines, stress signaling, growth factors, fibrosis, and myocyte loss. There is now evidence that autophagy plays an important role as well [36–39].

Kanamori et al. [36, 40] used a mouse myocardial infarction model to investigate the function of autophagy in ischemic remodeling. The dynamics were assessed during the subacute and chronic stages (1, 2 and 3 weeks) after coronary occlusion. The effects of agents that alter the autophagic process on the progression of post-infarction remodeling and left ventricular function were studied as well. These investigators observed an increase in autophagic activity over time and a unique morphology and localization that was dependent on the stage of infarction. During the subacute stage, autophagy was more prominent in the border zone than in the more remote areas. In the chronic stage, autophagic activity was greater in the remote areas. The investigators speculate that the changing distribution of autophagic activity is related to the progression of cardiac remodeling and are a reflection of changes in wall stress. When bafilomycin A1 was used to block autophagy, cardiac dysfunction was exacerbated; stimulation of autophagy with rapamycin mitigated the cardiac dysfunction and remodeling.

These findings are consistent with those reported by Buss et al. [41, 42] who used a rat infarction model. In this study, everolimus (RAD) was used to inhibit mTOR; RAD treatment 1 day after the MI resulted in reduced ischemic remodeling and improved LV function. The effects were observed even when the animals were treated 3 days after the MI. Sustained improvement of LV function was observed 3 months after MI even when RAD treatment was discontinued after 1 month. Collectively, these findings and reports by others support the concept that autophagy is an adaptive process that protects against adverse post-ischemic remodeling.

Indirect evidence that autophagy plays a significant role in protection against post-myocardial infarction remodeling is found in preclinical studies examining the effects of intermittent fasting (IF) and statins. After 3 months IF in rats, Ahmet et al. [43, 44] induced MI in rats by permanent coronary artery occlusion. In the IF cohort, infarct size after 24 h was twofold smaller, the number of apoptotic myocytes was fourfold less, and the inflammatory response was reduced compared to rats with regular every day feeding. Serial echocardiography that 10 weeks after MI left ventricular (LV) remodeling and infarct expansion were absent in the IF group. In a cohort of animals with similar MI size at 1 week, there was less remodeling, better LV function, and no MI expansion. They concluded that IF protects the heart from ischemic injury and attenuates post-MI cardiac remodeling through anti-apoptotic and anti-inflammatory mechanisms. Although not emphasized by this group, it is important to note that intermittent fasting and caloric restriction (starvation) are potent inducers of both autophagy and mitophagy.

With respect to statins, while 3-hydroxy-3-methylglutaryl coenzyme A (HMG-CoA) inhibitors are known to reduce morbidity and mortality in patients with ischemic heart disease, it is controversial whether they are effective in reducing major cardiovascular events in patients with established heart failure. In the CORONA and GISSI-HF trials, rosuvastatin therapy (10 mg daily) was not associated with a reduction in major cardiovascular events [45, 46]. The findings strongly suggest that routine statin therapy in heart failure patients is not warranted. It is important to note, however, that (a) it is unknown whether higher doses would have been beneficial and (b) in the CORONA trial, statin therapy was associated with significantly fewer hospitalizations. Interestingly, McMurray et al. [47], in a retrospective study of the CORONA patients, reported that rosuvastatin was associated with significantly better outcomes in patients with hs-CRP ≥2.0 mg/L. Independent of these findings, there is experimental evidence that statin therapy may reverse pathologic ventricular modeling [48].

Hayashidani et al. [49] using a mouse myocardial infarction model reported that fluvastatin, when added to the drinking water for four weeks, increased survival from 61 to 86 %. Serial echocardiograms revealed a reduction in chamber dilatation and hypertrophy and improvement in ventricular function. Treatment was also associated with an attenuation of the increase in metalloproteinases that was observed in the untreated group. These observations are consistent with and extend the findings by Lefer et al. [50] who reported that statins are cardioprotective in the setting of ischemia/reperfusion injury. Given the recent findings in the Gottlieb laboratory [51] that statins exert their cardioprotective properties through upregulation of autophagy and mitophagy, it is possible that patients at risk of adverse post-infarction remodeling might benefit from drugs that target the machinery of autophagy and upregulate autophagic flux.

In general, increasing cell size is mediated by mTOR, which suppresses autophagy. In contrast, autophagy participates in reducing cell mass and is generally considered to be anti-hypertrophic. However, the energetic cost of autophagy and the need to continually replace cellular constituents may be too high a price to pay in a fragile, energy-restricted heart. Thus, it is conceivable that uncontrolled autophagy may contribute to the demise of cells that are unable to counterbalance catabolism with biosynthesis.

### Load-induced remodeling

The initial response to pressure overload in the heart is an increase in muscle mass to increase the force of contraction; the result is left ventricular hypertrophy. As with ischemic remodeling, autophagy has been implicated in playing an important role. There is, however, controversy as to autophagic activation is beneficial or deleterious. To investigate the role of autophagy, Zhu et al. [16] studied mice subjected to surgical constriction of the proximal aorta (severe thoracic aortic banding [sTAB]). At 24 h and 1 week, the mortality (<10 %) was comparable to conventional thoracic aortic constriction (TAC) but function indicators demonstrated a rapid onset of clinical heart failure. The latter was associated with a marked increase in the number of autophagosomes that peaked at 48 h and persisted for at least 3 weeks. In mice subjected to sTAB that were haploinsufficient for Beclin 1, a protein required for early autophagosome formation, autophagosome abundance was blunted and pathologic remodeling was diminished. Conversely, Beclin 1 overexpression increased both autophagosome abundance and the severity of remodeling. The investigators concluded that pressure overload triggers cardiac autophagy, and this response contributes to progression of heart failure. However, as noted above, autophagosome abundance could be a sign of impaired flux, which will increase inflammatory signaling via the TLR9 and inflammasome pathways [22, 23].

In contrast, Nakai et al. [33] reported that activation of autophagy is beneficial. Cardiac-specific deletion of Atg5, a rate-limiting protein involved in autophagosome initiation, resulted in cardiac hypertrophy, ventricular dilation, and contractile dysfunction. Moreover, pressure overload in these Atg5 mutant animals triggered a rapid and dramatic decline in cardiac function. Ultrastructural analysis showed disorganized sarcomere structure and mitochondrial misalignment and aggregation. While cardiac-specific deficiency of Atg early in cardiogenesis showed no such cardiac phenotypes under baseline conditions, 1 week after TAC the animals developed ventricular dysfunction and dilatation. The investigators concluded that basal autophagy is required for normal cardiac function and upregulation of autophagy is an adaptive response to hemodynamic stress. Similar findings have been reported by other investigators, albeit more indirectly [39].

The inconsistent conclusions regarding the role of autophagy in overload remodeling is related to the model studied. Rothermel and Hill [52] have suggested that Atg5 deficient mice have near-complete loss of constitutive autophagy while the Beclin 1 ^+/−^ mice have only a 50 % reduction. As a consequence, functions carried out by basal levels of autophagy are lost in the Atg5-deficient hearts but not in the Beclin 1 ^+/−^ hearts. They suggest that this explains why WT mice, in the setting of pressure overload, are able to mount a robust autophagic response that is maladaptive. A more likely explanation, however, rests with the findings by Ma et al. [15] discussed earlier in the section on cardioprotection, namely, that Beclin 1 inhibits autophagic flux: (a) with less clearance there is less protection against remodeling, (b) in the Beclin 1 ^+/−^ mice, there is less inhibition of autophagic flux; hence, there is less adverse remodeling. If the findings by Diwan’s group are confirmed by other laboratories, then these two seemingly contradictory observations can be reconciled without having to implicate a threshold for autophagy, whereby it is adaptive in the same setting under one set of conditions and maladaptive in another. If autophagy is indeed principally an adaptive process, this would suggest that maladaptive autophagy is more likely a reflection of dysfunctional autophagy.

Indirect evidence that autophagy plays a role in moderating hypertrophy is the study by Finckenberg et al. To evaluate the role of caloric restriction (CR), this group used 4-week-old double transgenic rats (dTGR) with human renin and angiotensinogen genes. The objective was to test the hypothesis that CR would have a salutary effect on survival. In the CR animals, angiotensin II (Ang II)-induced mitochondrial remodeling and cardiac hypertrophy via blood pressure-independent mechanisms were ameliorated. These changes were associated with increased endoplasmic reticulum stress signaling and autophagy. The end result was superior survival; the mortality was 85 % in the unrestricted diet dTGRs and 25 % in the CR cohort at 8 weeks. The investigators concluded that CR, in this setting, affords protection against cardiac hypertrophy and death. However, CR may confer other benefits independent of autophagy.

In summary, the role of autophagy in the modulation of load-induced remodeling is controversial. The issue, however, is not whether it is important, but rather whether activation of the autophagic machinery is beneficial or deleterious. Independent of the controversy, it appears that sufficient evidence is available to warrant the development of pharmacological agents that are designed to target the autophagy process in an effort to prevent and treat load-induced adverse remodeling.

### Desmin-related cardiomyopathy (DRCM)

DRCM is a cardiomyopathy caused by a missense mutation in the αB-crystallin (CryAB) gene and is characterized by accumulation of misfolded proteins. To assess whether accumulation of aggregate-prone protein affects autophagic activity, Tannous et al. [53] infected neonatal rat ventricular myocytes (NRVMs) with virus containing WT human CryAB (Ad-CryAB^WT^), virus expressing mutant human CryAB^R120G^ or empty virus. Mutant CryAB^R120G^ triggered a >twofold increase in autophagic activity. Overexpression of CryAB^R120G^ induced aggregate accumulation and systolic heart failure. Autophagic activity was detected at 2 months well before there was evidence of a decline in cardiac function which was evident at 12 months. When CryAB^R120G^ mice were crossed with Beclin 1 ^+/−^ mice, autophagy was blunted and heart failure progression increased. This was associated with an acceleration of ventricular dysfunction and early mortality. These findings suggest that activation of autophagy in this setting is beneficial in attenuating progression of protein misfolding cardiomyopathies.

### Lysosomal storage diseases

Danon disease is a glycogen storage disease that may present as a cardiomyopathy. It is characterized by defective autophagosome-lysosome fusion due to a mutation in the lysosomal membrane receptor LAMP2 (lysosome-associated membrane protein 2). Deficiency of this autophagy gene results cardiomyopathy, myopathy, and variable intellectual disability; pathologically the patients have intracytoplasmic vacuoles containing autophagic material and glycogen in skeletal and cardiac muscle cells. The accumulation of autophagosomes in this glycogen storage disease is consistent with a defect in autophagosome clearance, that is, impairment in autophagic flux. Given that impaired autophagic clearance is a hallmark of the disease, a therapeutic approach might be the use of pharmacological agents to inhibit the initiation of autophagy in order to reduce the autophagosomal burden [54].

Fabry disease is a lysosomal storage disorder caused by a deficiency in α-galactosidase A that results in progressive accumulation of neutral glycosphingolipids. It is associated with hypertrophic cardiomyopathy that is responsive to enzyme replacement therapy [55]. Recently, Fabry cells were shown to exhibit impaired autophagic flux [56].

Pompe disease, is a lysosomal storage disorder caused by deficiency of acid alphaglucosidase, resulting in massive accumulation of glycogen in cardiac and skeletal muscle, leading to progressive dysfunction. Enzyme replacement is more effective for ameliorating cardiac hypertrophy than for skeletal muscle. Recently, it was reported that suppressing autophagy improved the efficacy of enzyme replacement in a mouse model of the disorder [57].

What these diseases of lysosomal function reveal is that impaired autophagic flux is as bad (and perhaps worse) for the heart as insufficient autophagy. Lysosomal storage diseases, in which the dominant feature is accumulation of glycogen or glycosphingolipids, may carry a second mode of injury in which degradation of other cellular constituents (such as mitochondria) may lead to activation of inflammatory pathways. The severe consequences of pressure overload in mice lacking the lysosomal enzyme DNase II, required to degrade mitochondrial DNA [24], illustrate the inflammatory consequences of lysosomal dysfunction leading to interaction of nondegraded mitochondrial DNA with TLR9 or the inflammasome.

## Autophagy and heart failure

In contrast to remodeling, the role of autophagy in heart failure is less clear. While autophagic activation may protect the heart from failing, there are reports that autophagy is associated with the progressive destruction of cardiomyocytes in the failing heart; the implication being that autophagy causes failure. Shimomura et al. [58] examined heart tissue resected during partial left ventriculectomy in 27 patients with idiopathic dilated cardiomyopathy. Electron microscopy revealed degenerated mitochondria and myofibrillar lysis. Large vacuoles were surrounded by a single membrane and occasionally contained intracytoplasmic organelles which suggested that they were autophagic vacuoles. On the basis of these findings, it was suggested that autophagic degeneration may represent one of the underlying mechanisms of heart failure. What was not addressed was the presence or absence of double membrane structures so characteristic of viable autophagosomes.

To explore this possibility, Miyata et al. [59] studied the UM-X7.1 hamster model of human dilated cardiomyopathy. In this model, heart failure develops progressively and the mortality rate is 50 % at 30 weeks of age. Ultrastructural analysis of these hearts revealed that many of the cardiomyocytes contained autophagy vacuoles and degraded mitochondria. Immunoassays showed that most of the cardiomyocytes with a leaky plasma membrane were positive for cathepsin D. Based on these findings, it was concluded that there is a causative link between autophagic activity and cell death. In a separate series of experiments, UM-X7.1 hamsters were treated with granulocyte colony-stimulating factor (GCSF). The rational was based on reports that GCSF is a regeneration- and/or repair-related cytokine. Treatment resulted in a marked improvement in survival, ventricular function, and remodeling. This paralleled a reduction in myocardial fibrosis and autophagy. Since there was no evidence of transdifferentiation from bone marrow cells into cardiomyocytes, it was concluded that the beneficial effects of GCSF were due to an anti-autophagic mechanism.

This study is similar to other studies that rely primarily on electron microscopy to establish a link, positively or negatively, between autophagic activity and myocardial remodeling. Ultrastructural analyses cannot ascertain whether the presence of autophagosomes reflect a cause of injury or, in fact, represent a targeted response to injury. And, as noted earlier, autophagic flux needs to be taken into account. In the absence of flux measurements, one could mistakenly conclude that autophagy is the cause of heart failure in Danon disease as opposed to impaired autophagy.

An intriguing report by Kassiotis et al. [38] supports the view that autophagy is an adaptive mechanism in the failing human heart. They studied tissue samples from 9 patients diagnosed with dilated cardiomyopathy who were placed on a left ventricular assist device for end stage heart disease. Left ventricular tissue was obtained from the apex during implantation of the device and upon explantation of the device at the time of transplantation. To determine how levels of autophagy were influenced by mechanical unloading, autophagy markers were measured prior to and following unloading. In the unloaded failing heart, the Atg5-Atg12 conjugate, Beclin 1, and LC3-II were all decreased. Because a number of signaling pathways that that regulate both autophagy and apoptosis overlap, apoptotic markers were also assessed pre- and post-mechanical unloading [60]. In this study, there was no significant difference in Bcl-2 levels or TUNEL-positive nuclei between groups. Although the effect of unloading on apoptosis was unclear, the investigators concluded that mechanical unloading decreases autophagy and autophagy is an adaptive mechanism in the failing heart.

## Conclusion

**Fig. 1:**
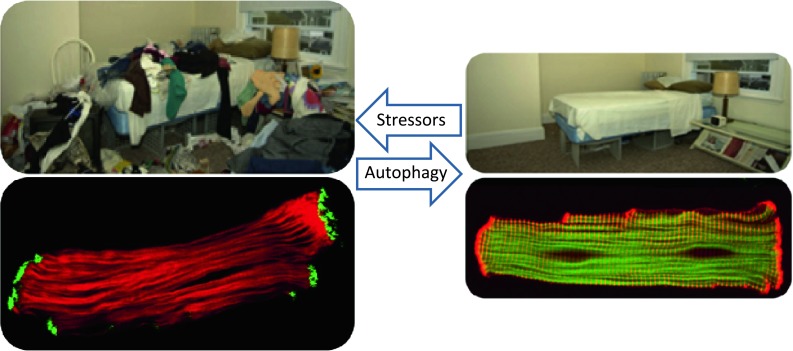
Stressors such as pressure overload drive the hypertrophic response, resulting in accumulation of damaged organelles and protein aggregates. Autophagy defends against cellular clutter

Autophagy and mitophagy play an important role in the modulation of ischemia–reperfusion injury, remodeling, and heart failure. While controversy exists as to whether this is a salutary or deleterious role, the preponderance of evidence suggests that autophagy and mitophagy are important protective mechanisms across the spectrum of ischemic injury. Autophagy is a means to de-clutter the cell and restore functionality (Fig. 1). It is likely that dysfunctional or impaired autophagy contributes to the morbidity and mortality associated with cardiovascular disease. For autophagy to move fully into the therapeutic arena, however, better tools are needed to facilitate the discrimination between functional autophagy and dysfunctional and/or impaired autophagy, as well as to image autophagy in vivo. Likewise, a better understanding of the functionality of autophagy and interplay between mitophagy and mitochondrial biogenesis is needed. Both could open the door to the development of novel effective therapies for the prevention and treatment of ischemia–reperfusion injury, pathological remodeling, and heart failure in humans.
